# Beyond Traditional TOD: Integrating Multiuse Paths and Bike Share into Public Transit to Address the First/Last Mile Issue

**DOI:** 10.1007/s40864-022-00182-x

**Published:** 2022-12-05

**Authors:** William P. Rogers, Na Chen, Johanna W. Looye

**Affiliations:** 1 School of Planning, College of Design, Architecture, Art, and Planning, University of Cincinnati, 346 Clifton Court, Cincinnati, OH 45221-0016 USA; 2 Center for Chinese Public Administration Research, School of Government, Sun Yat-sen University, No. 135, Xingang Xi Road, Guangzhou, 510275 People’s Republic of China

**Keywords:** Bike share, Multiuse paths, Public transit, First/last mile, Integration

## Abstract

Transit-oriented development (TOD) has been promoted worldwide as an integrated land-use and transportation strategy to foster urban sustainability. Bike share provides people with a convenient and relatively affordable way to enlarge the spatial scale of TODs across urban communities, as a solution to the first/last mile (FLM) issue with respect to the transit nodes of TODs. Even though barriers to FLM have been frequently studied, few studies incorporate people’s perceptions of their barriers and/or the integration of multiuse paths (MUPs) into the network of bike share and public transit. Using a survey conducted in the Greater Cincinnati area, Ohio, this study aimed to answer the following questions: (1) What are people's major barriers to integrating different green transportation modes and/or facilities (bike share, MUPs, public transit)? (2) To what extent does the built environment around people’s residential location affect their integration level of MUPs, bike share, and public transit? (3) Which improvements would most likely encourage people to integrate them more often? With descriptive statistics, spatial analysis, and statistical comparison, we found that (1) the major barrier to integrating MUPs into the green transportation system was their lack of connection and availability to transit and bike share; (2) a person’s living environment is spatially related to whether a person integrates bike share; and (3) more respondents would use MUPs more often if an integrated green transportation system could be provided or improved. These findings suggest the potential of incorporating MUPs and bike share into TOD strategies to address the FLM issue.

## Introduction

Bike share, multiuse paths (MUPs), and transit are each growing at various rates and are overcoming various challenges driven by popular demand for greenways and for more ways to explore their cities. Bike share has not only grown increasingly popular but also has seen drastic changes in design, functionality, and more importantly in its connections to other transportation modes. Replacing short, motorized vehicle trips with bike share trips helps reduce carbon emissions, promote a healthy mental and physical life, and strengthen the local economy. While this is clearly possible for short trips, they are not the only way bike share can be used. For example, the function of bike share as a mode to complete part of the tour with multiple transportation modes has been widely recognized. MUPs are becoming an amenity for people to get exercise and as safe paths for travel in daily life [1]. However, the discussion of MUPs as a solution to the first/last mile (FLM) issue for public transit is still limited, especially as compared to the ones over bike share. Moreover, existing studies rarely shed light upon their connections and functions from the perspective of transit-oriented development (TOD). TOD has been promoted worldwide as an integrated land-use and transportation strategy to bridge the FLM gap and thus foster urban transportation sustainability [2]. While bike share has been studied for its role in TOD as a convenient and relatively affordable way to enlarge the spatial scale of TODs across urban communities, the connection role of MUPs in this system is underestimated [3, 4].

This study hypothesizes that the extent to which people are positively and strongly familiar and comfortable with integrating bike share stations, transit stops, and MUPs affects their confidence in overcoming FLM barriers through bike share. We examine this through a mix of statistical and spatial analysis based on three research questions. First, what are people's major barriers to integrating different green transportation modes and/or facilities (bike share, MUPs, public transit)? Second, to what extent does the built environment around people’s residential location affect their tendency to integrate public transit, bike share and MUPs (i.e., using two or more modes one after the other, or using bike share on a MUP)? Finally, which improvements would most likely encourage people to integrate these green transportation modes more often?

Research on FLM has focused considerably on the bicycle road network, distribution of transit stations, distribution of bike share stations, integration with different forms of transit, and various types of barriers to increasing bike share ridership [5–7]. This study contributes to the literature in two important ways. First, a local MUP network is incorporated into the integration analysis to determine how it can contribute to the FLM solution. Second, in addition to collecting stated survey data about barriers to using bike share and MUPs, this study collects revealed behavior data about barriers to using bike share and MUPs. The results of this study can be used by bike share programs, planners, researchers, and policy makers to take advantage of the existing green transportation infrastructure by increasing their integration.

## Literature Review

### Challenges in Planning for Bike Share

Bike share has progressed from chaotic beginnings to formal, large, technology-focused parts of the transportation networks in cities. The primary benefits are commonly summarized as protecting the environment, improving personal mental and physical health, and social equity [8–11]. Despite this, transportation planners face a series of challenges to implement bike share. The major challenge is the inclusion of multimodal planning as a larger function of local, regional, and state planning efforts within the transportation planning and engineering efforts for the entire population. These efforts need to coordinate the locations of bike parking and bike share facilities, transit locations, and daily destinations for the groups who use bike share or want to do so, and especially for those use bike share out of necessity [12]. More specifically, persons of color and/or low-income individuals cite more barriers to bike share than higher-income white residents [13], even though they are also the fastest growing group of riders [14].

Theoretically, TOD provides an appropriate community-based environment in terms of an integrated land-use (e.g., high density and mixed use) and transportation (e.g., transit) system to address the planning challenge faced by the maximization of those bike share benefits mentioned above. Vice versa, the role of bike share in TOD is specified as the increased active travel possibilities and enlarged catchment area from the transit nodes, particularly as compared to the walking mode [3].

### First/Last Mile Solution: Bike Share

Within multimodal planning, addressing FLM introduces a glue that strengthens the connections between various modes. If multimodal programs are to succeed in cities, they must recognize the opportunity and obligation to address the FLM issue at the onset. The promotion of these connections requires a good understanding of the benefits and barriers to the integration.

Cheng and Lin [15]’s study is noteworthy for its focus on people’s perceptions regarding the connection between metro transit and bike share. Their multinomial logit model revealed that the distance from the transit station to the bike share station had a significant negative effect and previous use of a station had a significant positive effect on bike share usage, as the way to continue their journey from a transit station. The case study’s cost-benefit analysis also showed the positive impact bike share programs can have in their extension of transit networks. However, the study lacked data on why, exactly, people are willing to make their choice and what prevents others who are not willing from making the same choice.

Designing transit and bike share stations together can be adopted when an entire network is expanded, and thus financial savings can be increased [16, 17]. Increased bike share usage can help bus ridership and has been predicted to reduce bus travel times by six minutes in Helsinki [18]. Bike share trips can generate transit trips and vice versa if bike share stations and bus stops are close enough [16]. In addition, incorporating bike share programs with TOD can increase its effectiveness. With less time spent on wayfinding and/or walking to the next transportation mode, concentrated bike share stations near TOD can translate to a seamless and quick transition when switching modes [15, 19, 20]. Another significant benefit of integrating bike share and transit service is the improvement of social equity in terms of enhancing low-income people’s quality of life through providing more transportation options [21].

While the benefits reviewed above are attractive for promoting the integration of bike share as a FLM solution, there are still many factors that influence the modal integration, such as weather, built environment and land-use, public transportation, station level, sociodemographic effects, and temporal factors and safety [22]. Severe weather is one widely recognized barrier to using bike share. For example, temperature, both hot and cold, can significantly affect cycling ridership [22]. To minimize this negative effect, bike share programs can take advantage of the weather conditions that their users are most comfortable with. For example, if people are not willing to bike under very cold temperatures, a bike share organization might focus its resources on the times when it is above a specified temperature [22]. Regarding the impact of the built environment, a strong positive correlation between the distribution and quality of bicycle infrastructure and bike share ridership has been found [23]. Specifically, a common barrier to using bike share is the difficulty of reaching the first transit station within a reasonable walking distance [24]. If an urban environment has low housing density, bike share stations will be near fewer people and walking to one will take longer. Similarly, the distance from other forms of transportation can be a factor that prevents people from combining bike share and transit [25–27]. Facilities grouped around a transit hub, however, make it easier to switch between modes and stay informed of availability. One of many suggested solutions is to inform riders through a website/app prediction on future availabilities of other forms of transportation [28].

Spatial inequity creates another barrier for many socially disadvantaged people to move around and access services like bike share for a higher quality of life. According to Eren and Uz [22], “Research investigating the relationship between social inequality and low station access has shown that [bike share] stations are often established in areas with high income and densely populated areas, tourist sites, city centers, and recreation areas.” In other words, disadvantaged people cannot even live near bike share stations.

### First/Last Mile Solution: Multiuse Paths

The relationship between MUPs use and transit connectivity is limited. Based on a trail intercept survey conducted on the MUPs in the Greater Cincinnati region by Tri-State Trails and Interact for Health, Chen et al. [1] developed ordinary probit models and found that within the utilitarian cyclist population, there was a higher likelihood that the purpose of the trip was utilitarian if the distance from an urban trail to the nearest bus stop was shorter. Further, a significant and positive relationship was found between the distance to bike share stations from trails for utilitarian trail users, while marginal opposite relationship found for recreational trail users.

After analyzing a survey of people served by the Utah Transit Authority with an Importance-Satisfaction analysis and a path analysis, Park et al. [29] provided practical suggestions to improve public transit riders’ satisfaction and loyalty, especially their experience on the FLM problem. One suggestion is related to access/egress/transfer comfort improvements, such as the connectivity of sidewalks and protected bike lanes which are part of the MUPs system. However, the importance of these path facilities is mostly marginally studied as compared to other factors.

TOD is supported by the accessibility people have to it. MUPs and bike share programs offer the potential to make TOD more valuable by increasing the accessibility. Our study attempts to address this concern by asking people through survey questions about their behaviors and barriers to changing them.

## Methodology

To answer the research questions described above, we conducted a bike share survey from October 1, 2020 to October 31, 2020, using Qualtrics to collect data on travel behavior and perceptions on bike share and MUPs and their integration with public transit. Questions asked people about their demographics, current use of MUPs, bike share, transit, and if and how they use them together or after one another. For example, it was asked if bike share is used before or after taking the bus. Questions from the bike share non-profit, Cincy Red Bike who sent out the survey to its registered members, were included to help understand what would encourage people to use bike share more. With the survey data, statistical and spatial analysis methods were combined to discover how people’s integration of green transportation modes varies across different socioeconomic groups, perceptions, and the built environment.

### Study Region and Data

**Fig. 1:**
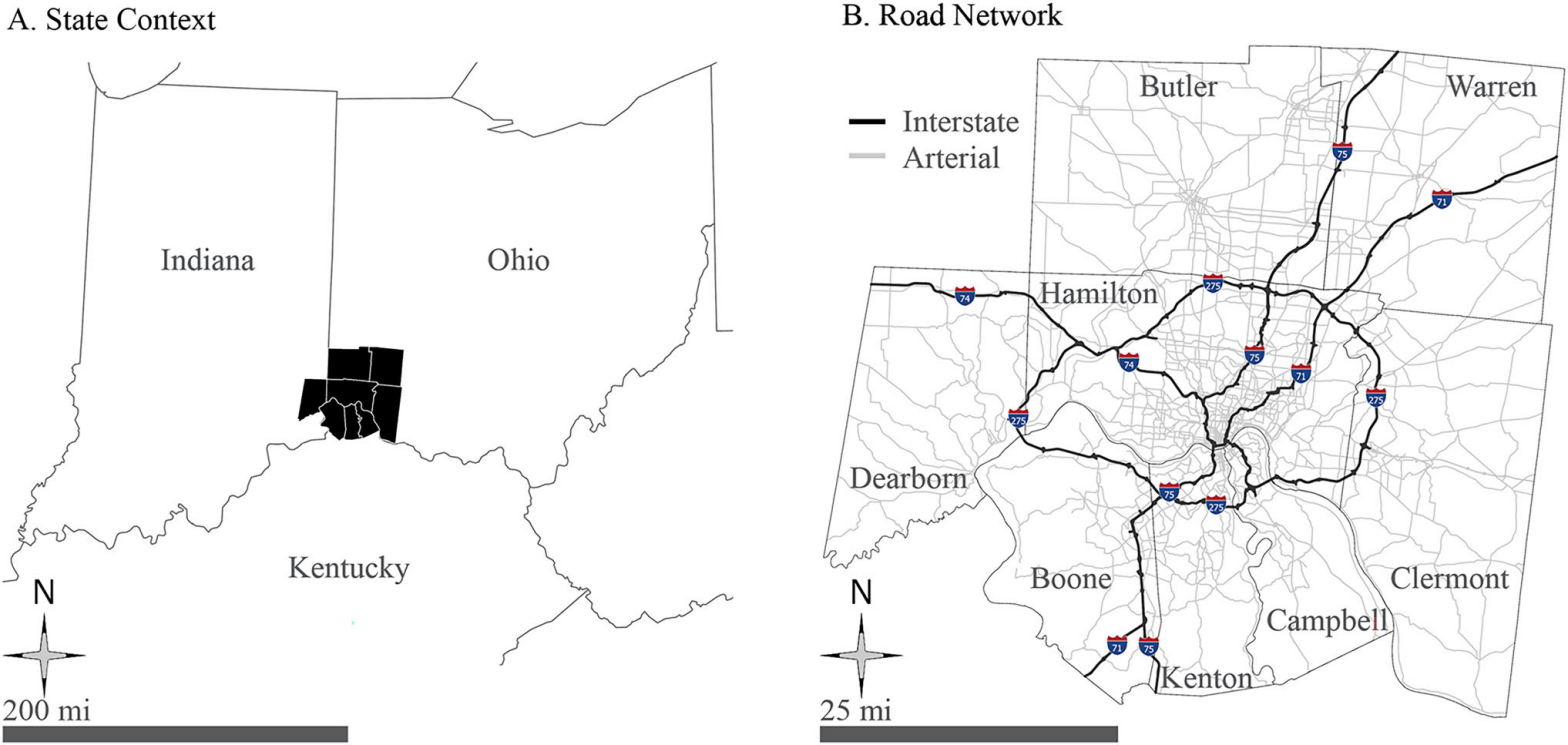
The Greater Cincinnati study region

The eight-county region named “OKI” is at the intersection of Ohio, Kentucky, and Indiana surrounding Cincinnati (see Fig. 1). This area, named Greater Cincinnati, was chosen for its medium size and potential for promoting cycling culture and public infrastructure investment. The OKI Metropolitan Planning Organization (MPO) oversees the planning efforts and works with the Southwest Ohio Regional Transit Authority and the Transit Authority of Northern Kentucky for transit planning. The streetcar (the Cincinnati Bell Connector) in Downtown Cincinnati has become a great addition to the public transit system in the region. As of 2021, Cincy Red Bike had nearly 60 bike share stations and more than 500 bikes across the Greater Cincinnati area. Red Bike is a crucial green transportation option for the Greater Cincinnati area, not only for tourism and recreation, but also in helping solve the FLM issue for people commuting to/from work and for other purposes, especially for those people living near the University of Cincinnati campus and Downtown Cincinnati. As these expand in both spatial and temporal coverage throughout the week and day, successfully addressing the FLM issue will provide more opportunities to improve people’s quality of life in Greater Cincinnati.

The survey results include 415 responses of which 245 provided valid residential locations which were geocoded with ArcGIS Pro. U.S. Census block groups were chosen as a proxy for neighborhood due to the shorter length of trips that cyclists take as compared to traffic analysis zones (TAZ) or census tracts and also due to the relatively better data availability at this level. Four modes of transportation were reported to create an integration level for each respondent, which is specifically described in the following sections.

### Statistical Analysis

The three research questions focus on the integration of bike share, MUPs, and public transit. Therefore, this study identified two groups of respondents from the survey results: Integration Group and No Integration Group. Respondents who reported that they have integrated bike share and bus and/or bike share and MUPs were classified into the Integration Group. Respondents who had not reported integrating any combination of these three modes were classified into the No Integration Group. Descriptive statistics and statistical comparison using *χ*^2^ tests were then conducted to identify the characteristics of these two groups and their statistical differences.

### Spatial Analysis

Spatial analysis investigated the spatial relationship between survey participants and their home’s surrounding built environment with the following steps. First, participants’ residences were geocoded with the nearest intersection to their home, ZIP code, and city in ArcGIS Pro. Second, these locations were spatially compared to the facilities of four green transportation modes (bike share stations, bus stops, streetcar stops, and MUPs) in terms of 400-meter buffering from these facilities. Four hundred meters, or sometimes a quarter of a mile, is a commonly used distance in pedestrian transportation planning, as beyond this distance, people are found to be less likely to walk to a destination in an urban setting [30, 31]. These were then analyzed with a *χ*^2^ test to determine their difference by integration level. Additionally, five built environment variables (intersection density, population density, proportion of single detached homes, employment density, and land-use entropy) were used to classify the block groups in the study region into five neighborhood types using a *K*-means clustering approach which has been widely applied for better capturing the comprehensive characteristics of the built environment [32, 33]. The clustering results were then compared by the two integration groups with a *χ*^2^ test to understand the connection between the neighborhood types and their residents’ integration situation.

## Results

Based on the three research questions for the integration level, details of results are presented and discussed in this order: (1) descriptive statistics and statistical comparison, and (2) geographical mapping and spatial analysis. The integration level is specifically defined as two groups that were created based on the survey results: “Integration Group” (those who have used at least two green transportation modes together in one journey) and “No Integration Group” (those who have not). The sizes of the two groups created for statistical analysis are as follows: 91 Integration (30.2% of 301 responses) and 210 No Integration (69.8%). Participants in the Integration Group have used bike share and/or MUPs before, after, or both before and after a transit trip. There were 24 submissions in Qualtrics which received no data on any questions, leaving 391 responses with at least one question answered and 301 valid responses. Therefore, depending on responses, the “*n*” will vary from question to question in the tables throughout this section.

### Descriptive Statistics and Statistical Comparison Results

Respondents’ sociodemographic characteristics, attitudes towards bike share use, and attitudes towards integrating green modes or facilities like MUPs, were analyzed with descriptive statistics and *χ*^2^ test. Reported barriers to integrating green transportation modes and facilities were also analyzed for the two groups.

#### Sociodemographic Characteristics

Table 1 provides the results of some key sociodemographic variables from the survey across the two integration groups. The age of 273 participants is listed by generation. The mean age of the No Integration Group (35.8) is less than that of the Integration Group (40.5). Many of the young millennials are still in school and/or live at home with parents and therefore have not explored other forms of transit. Many of them likely use personal vehicles and need the bus less often or simply do not use a vehicle at all. Males are the majority in both groups. Males being the majority of surveyed bike share users have been found in other studies with similar reason for standard bicycles. In the U.S., cycling—a potentially dangerous activity without proper infrastructure—has traditionally been a male sport [34] and males are known to take higher accident risks than females [35]. This leads to a disproportionate number of males riding any kind of bicycle.

**Table 1:** Group comparison by sociodemographic characteristics (*χ*^2^ tests)

		No integration		Integration		*p value
		*n*	% within group	*n*	% within group	
Age**	Total	189	100.0	84	100.0	
	Young millennials (aka Gen Z) (1995 to 2010)	68	36.0	16	19.1	**0.005**
	Mid-millennials (1989 to 1994)	30	15.9	9	10.7	0.261
	Older millennials (1979 to 1988)	26	13.8	19	22.6	0.069
	Gen X (1965 to 1978)	37	19.6	26	31.0	**0.039**
	Baby boomers (1946 to 1964)	28	14.8	14	16.7	0.695
Gender	Total	210	100.0	91	100.0	
	Female	101	48.1	28	30.8	**0.004**
	Male	103	49.1	61	67.0	**0.004**
	Non-binary	0	0.0	2	2.2	NA
	Prefer not to answer	6	2.9	0	0.0	NA
Race	Total	210	100.0	91	100.0	
	White alone	177	84.3	76	83.5	0.394
	Non-white alone	22	10.5	13	14.3	0.394
	Prefer not to answer	11	5.2	2	2.2	
Education	Total	199	100.0	91	100.0	
	Bachelor’s or higher	131	65.8	57	62.6	0.597
	Less than bachelor’s	68	34.2	34	37.4	0.597
Income	Total	127	100.0	57	100.0	
	$49,999 or less	51	40.2	27	47.4	0.564
	$50,000–$99,999	47	37.0	26	45.6	0.443
	$100,000–$199,999	61	48.0	22	38.6	0.190
	$200,000 or more	19	15.0	9	15.8	0.992

*Bold values indicate *p* value of 0.05 or less

**Age was collected as specific years and grouped into generations based on [27]

“White alone” consists of about 84% in both groups. Integration Group does have a higher percentage of nonwhite alone people at 14% while No Integration Group has about 10%, but the difference is not statistically significant. Although there is no significant difference between the groups in terms of education, percentages within groups do vary. “Bachelor’s or higher” comprises around 64% in both groups. There is a higher percentage of highly educated people within the Integration Group. This may imply that those who have a bachelor’s or higher degree are more concerned with curbing their automobile usage. Even when income levels are condensed down to four groups with the lowest level “$49,999 or less,” which includes Cincinnati’s median income of $40,640 and lower, there is no significant difference between groups. There is, however, a higher percentage of people who make $99,999 or less in the Integration Group than the No Integration Group, meaning that those with the time, energy, and resources choose multimodal transportation.

#### Travel Behavior and Attitudes

Table 2 presents results for attitudes on bike share use between the two groups. There are significant differences between the types of vehicles that people used within the 30 days prior to the survey. The more a person integrates green transportation modes, the less likely they use non-green transportation modes during the same time period. This is likely due to their familiarity with modes such as bicycling and/or the usage of bike share and scooter. Except for the bus, the people in the Integration Group tend to use modes of transportation that do not involve combustion engines (some Go Metro buses are hybrid). Personal vehicle use was higher in the No Integration Group at 46%, although it is only slightly statistically significant at the 0.1 level (*p* = 0.092). People who reported that they used bus pass (e.g., one-time use, monthly) are more likely to be in the Integration Group (8.7%).

**Table 2:** Attitudes on bike share use

		No integration		Integration		*p value
		*n*	% within group	*n*	% within group	
*Which of the following vehicles have you used in the past month? (Check all that apply)*						
	Total	558	100.00	299	100.00	
	Any bus (at least 1 chosen)	22	3.94	26	8.70	**0.000**
	Personal vehicle (e.g., car, van, SUV, truck)	258	46.24	75	25.08	0.092
	Personal bicycle (other than Red Bike)	76	13.62	55	18.39	**0.000**
	Any Red Bike membership (at least 1 chosen)	40	7.17	51	17.06	**0.000**
	Rideshare (e.g., Uber, Lyft, Zipcar)	34	6.09	17	5.69	0.096
	Motorcycle/moped	5	0.90	1	0.33	NA
	Electric scooter or skateboard (e.g., Bird, Lime)	12	2.15	15	5.02	**0.000**
	Other	90	16.13	56	18.73	**0.000**
	None of the above	21	3.76	3	1.00	0.172
*What aspects of Red Bike do you enjoy the most? (Check top 3 only)*						
	Total	420	100.00	254	100.00	
	Convenience (at least 1 chosen)	64	15.24	66	25.98	**0.000**
	It’s fun	39	9.29	34	13.39	**0.000**
	Good availability of bikes at stations	14	3.33	22	8.66	**0.000**
	Good location of stations (e.g., close to where I need)	35	8.33	33	12.99	**0.007**
	Well priced	10	2.38	11	4.33	0.166
	I enjoy helping to protect the environment	31	7.38	17	6.69	**0.000**
	It’s good for my fitness/health	40	9.52	35	13.78	**0.000**
	Biking is my preferred transportation choice	18	4.29	25	9.84	NA
	The kiosk/online experience is great	1	0.24	1	0.39	**0.000**
	I don’t use Red Bike	168	40.00	10	3.94	NA
*What prevents you from using Red Bike or using it more often? (Check top 3 only)*						
	Total:	500	100.00	211	100.00	**0.000**
Weather	Bad weather (at least 1: rain, snow or wind or temperature)	62	12.40	38	18.01	**0.004**
Safety	Safety: crime-related	12	2.40	9	4.27	0.095
	Safety: traffic-related	50	10.00	17	8.06	0.713
	Public health concerns (e.g., COVID-19)	26	5.20	18	8.53	**0.028**
Physical environment	Steep hills	44	8.80	13	6.16	0.418
Bike availability	Not enough Red Bikes (at least 1: standard or electric)	26	5.20	22	10.43	**0.002**
Built environment	Not enough bike lanes	45	9.00	30	14.22	**0.005**
	Stations are too spread out	17	3.40	5	2.37	0.628
	No stations at Origin-Destination	71	14.20	29	13.74	0.625
	Not enough stations close to bus stops	1	0.20	3	1.42	NA
Other	No shower at destination	8	1.60	4	1.90	NA
	No access to helmets	12	2.40	0	0.00	NA
	High cost	41	8.20	9	4.27	0.115
	Other	85	17.00	14	6.64	**0.001**

Note: In this table, “*n*” indicates number or responses (up to three per respondent), not number of respondents

*Bold values indicate *p* value of 0.05 or less

The results on travel behavior and related attitudes are crucial to understand what improvements would influence people’s likelihood to overcome their barriers but have not been found until now in Greater Cincinnati. Regarding people’s top three barriers to bike share use, respondents in the Integration Group were more likely to report factors such as bad weather, public health concerns (e.g., COVID-19), lack of available bikes at Red Bike stations, and insufficient bike lanes. People in the No Integration Group were more likely to cite factors such as “no stations at Origin-Destination” and “steep hills.” These results are consistent with many studies [36, 37].

Regarding attitudes towards integration, people in the Integration Group were more likely to walk after taking a bus in a normal journey, but they were also more likely to take a different mode other than walking. Within the No Integration Group there were about 3% that cited taking a mode other than walking after the bus whereas almost 14% in the Integration Group reported using some other mode besides walking after taking the bus (see Table 3). The less respondents integrate bike share with transit or MUPs, the more likely they are to walk after a bus ride. When asked to rank different modes of transportation as their top preference after a bus ride, people in the Integration Group were more likely to select a mode of transportation other than walking like bike share or personal bike.

**Table 3:** Attitudes on integration

	No integration		Integration		*p value
	*n*	% within group	*n*	% within group	
*How do you normally continue your journey after you get off a bus?*					
Total:	243	100.0	94	100.0	
I never take the bus	136	56.0	28	29.8	**0.000**
Walk	100	41.2	53	56.4	**0.012**
Other (e.g., Red Bike, personal bike, electric scooter)	7	2.9	13	13.8	**0.000**
*Rank the following from 1 to 5: how you would prefer to continue your journey after getting off bus*					
Total:	98	100.0	66	100.0	**0.000**
Walk	82	83.7	33	50.0	**0.000**
Other	16	16.3	33	50.0	**0.000**
*What prevents you from using Red Bike when traveling to and from a bus stop? (Check top 3 only)*					
Total:	122	100.0	97	100.0	
Not enough Red Bike stations at my Origin-Destination	16	13.1	18	18.6	0.319
Not enough Red Bike stations at bus stops	7	5.7	15	15.5	**0.008**
Distance: Red Bike stations are too far away from my Origin-Destination	14	11.5	16	16.5	0.170
Distance: Red Bike stations are too far away from bus stops	9	7.4	5	5.2	0.601
Not enough regular Red Bikes	2	1.6	2	2.1	NA
Not enough electric-assist Red Bikes	4	3.3	10	10.3	**0.020**
Public health concerns (e.g., COVID-19)	8	6.6	4	4.1	0.513
Other	29	23.8	5	5.2	**0.000**
Nothing prevents me	33	27.0	22	22.7	0.666
*I would use the bus more often if Red Bike stations were integrated into more bus stops.*					
Total:	214	100.0	91	100.0	
Strongly agree	10	4.7	18	19.8	**0.000**
Somewhat agree	38	17.8	21	23.1	0.282
Neutral	101	47.2	32	35.2	0.053
Somewhat disagree	23	10.8	6	6.6	0.258
Strongly disagree	42	19.6	14	15.4	0.381
*I would use paths and trails more often if more Red Bike stations were along them*					
Total:	211	100.0	91	100.0	
Strongly agree	37	17.5	36	39.6	**0.000**
Somewhat agree	69	32.7	31	34.1	0.817
Neutral	63	29.9	16	17.6	**0.026**
Somewhat disagree	25	11.9	4	4.4	**0.044**
Strongly disagree	17	8.1	4	4.4	0.251
*I would ride the bus more often if there were more bike lanes, paths, and trails that led to bus stops*					
Total:	211	100.0	91	100.0	
Strongly agree	25	11.9	29	31.9	**0.000**
Somewhat agree	44	20.9	25	27.5	0.209
Neutral	81	38.4	18	19.8	**0.002**
Somewhat disagree	25	11.9	6	6.6	0.167
Strongly disagree	36	17.1	13	14.3	0.548
*Where do you normally use Red Bike? (Check top 3 only)*					
Total:	292	100.0	218	100.0	
On-road with traffic	55	18.8	58	26.6	**0.000**
On-road with “sharrow” (i.e., painted bike symbol on pavement)	21	7.2	26	11.9	**0.000**
On-road in a bike lane	36	12.33	37	17.0	**0.000**
On-road in a protected bike lane (e.g., Central Pkwy in Cincinnati)	16	5.5	25	11.5	**0.000**
Sidewalks (currently illegal in Cincinnati if over 15)	13	4.5	16	7.3	**0.002**
Multiuse paths (pedestrians and cyclists can both use these)	0	0.0	48	22.0	**0.000**
I don’t use Red Bike	149	51.0	7	3.2	**0.000**
Other	2	0.7	1	0.5	NA

*Bold values indicate *p* value of 0.05 or less

Barriers to integrating green transportation modes are different between the two groups. When asked of their barriers to Red Bike when traveling to and from a bus stop, people in the Integration Group were more likely to choose “not enough Red Bike stations at bus stops” or “not enough electric-assist Red Bikes.” People in the No Integration Group were more likely to choose “Public health concerns (e.g., COVID-19)” or “Other.” Three five-point Likert questions on integration were asked:

1. I would use the bus more often if Red Bike stations were integrated into more bus stops.
2. I would use paths and trails more often if more Red Bike stations were along them.
3. I would ride the bus more often if there were more bike lanes, paths, and trails that led to bus stops.

People in the Integration Group were more likely to “Strongly Agree” to each of these, but people in the No Integration Group also chose “Somewhat Agree” or “Strongly Agree” more than “Neutral” or any “Disagree” option. These results imply that measures and strategies to attract new riders should be planned differently from those taken to keep existing riders and members. New riders are less aware of how one might want to integrate modes and extend the range by using more than one mode. Existing users may have tried to integrate or want to do so but see the things that prevent it from happening.

### Geographical Mapping and Spatial Analysis Results

Overall, the geographical mapping and spatial analysis results indicate that the built environment significantly influences whether a person integrates green transportation modes or not. Responses with intersections reported were mapped to compare respondents’ locations with their surrounding built environment, in terms of buffering (400-meter) from the locations of bike share stations, bus stops, streetcar stops, and MUPs. Respondents were tabulated by the number of buffers they are located and their integration level. For example, around 32% of respondents within the Integration Group live within 2–4 buffers from those green transportation facilities. Table 4 shows that responses from the Integration Group are more likely to be within 400 meters of two to four selected green transportation modes (i.e., bike share, bus, streetcar, and MUP), indicating that the presence of green transportation alternatives affects a person’s chances of integrating them.

**Table 4:** Responses within buffers by integration group

No Integration			Integration		*p value
Within # of buffers	*n*	% within group	*n*	% within group	
Total	297	100.0	94	100.0	
0 to 1	232	78.1	64	68.1	**0.048**
2 to 4	65	21.9	30	31.9	**0.048**

*Bold values indicate *p* value of 0.05 or less

**Fig. 2:**
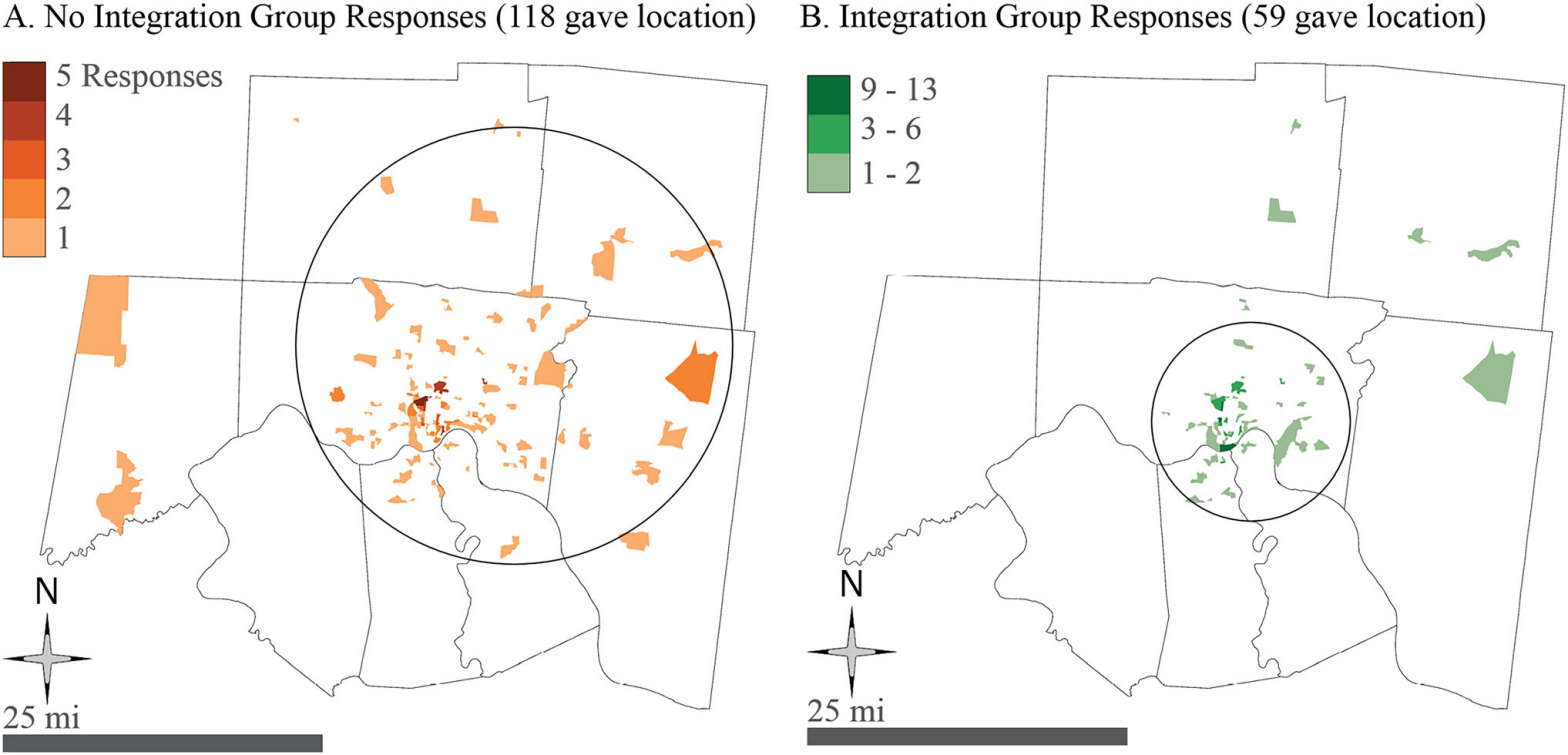
Integration Group quantities by location

Figure 2 shows respondents’ distribution across Greater Cincinnati. Most of them are in the urban core where population is most dense, but it appears that there is a difference in where the two groups live on average. As shown with the two circles in Fig. 2, in the Integration Group, these home locations are close within the urban core and the No Integration Group is much more dispersed. Based on geographical mapping, the closer a person lives to multiple green transportation facilities, the greater the chance of integrating them. The results from Table 4 and Fig. 2 to a certain extent echo with the recognition that one key factor for the level of integration is the availability of green transportation modes around the transit stops and other relevant facilities.

#### Built Environment

**Fig. 3:**
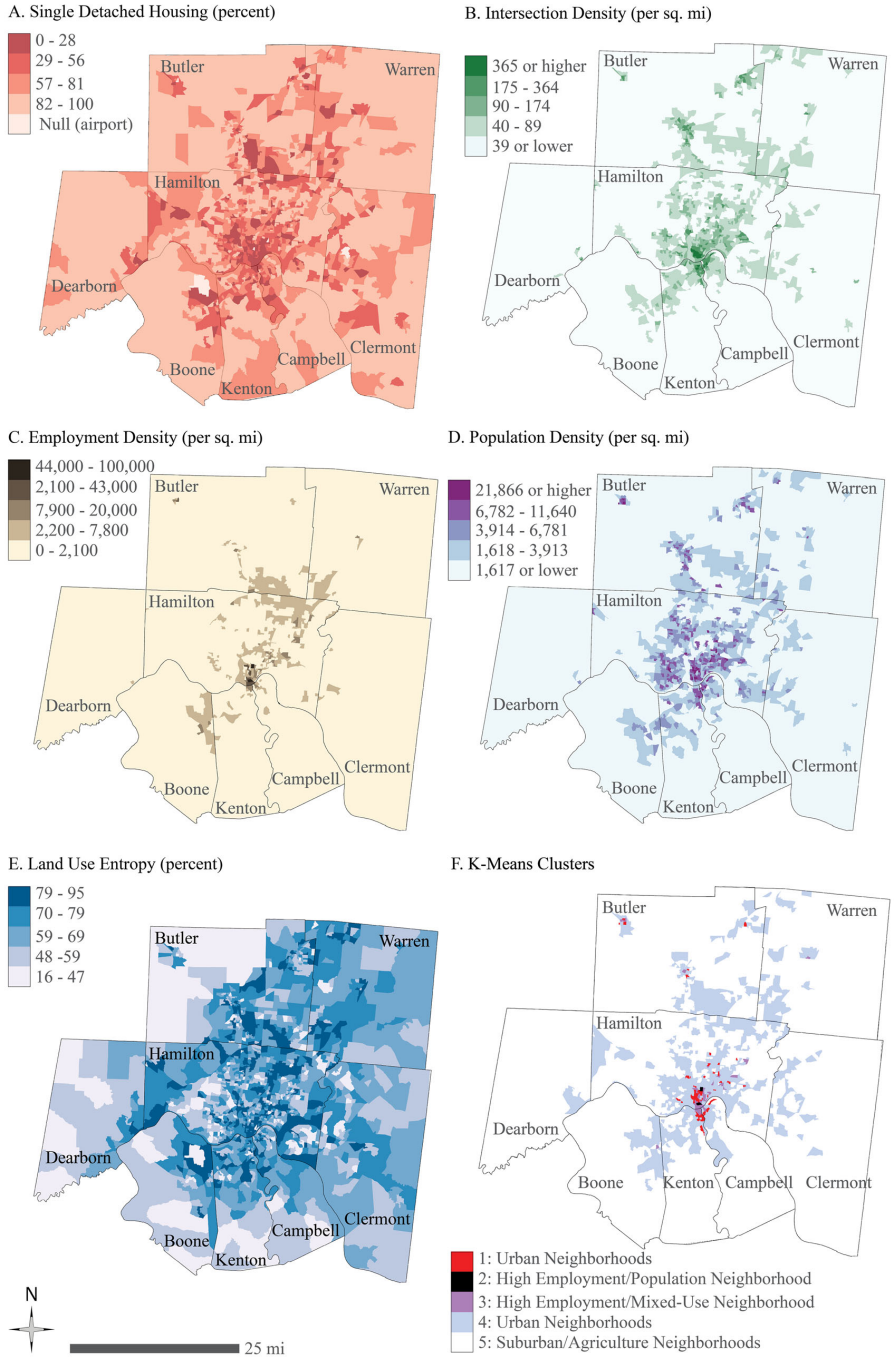
Built environment variables and clustering results

Five built environment variables were further selected to analyze their effects on a person’s tendency to integrate green transportation modes based on the literature review and data availability, including intersection density, population density, proportion of single detached homes, employment density, and land-use entropy (see Fig. 3A–E).

A *K*-means cluster analysis was applied to the five built environment variables to examine the relationship between the built environment and integration of green transportation (see Fig. 3F). Table 5 shows the clustering results sorted by population density. Cluster 1 is identified as urban neighborhood centers by its high densities in population, employment, and intersection. These block groups are primarily in urban residential and business districts of the study area. Cluster 2 has the highest densities in employment and intersection, the second-highest land-use entropy, and the lowest percentage of single detached housing, pointing to Downtown Cincinnati and the hospital areas around the University of Cincinnati. Cluster 3 is featured with the highest land-use entropy and the second-highest employment density. Cluster 4 covers mostly urban residential areas, and Cluster 5 refers to suburban areas with the highest percentage of single-family detached housing and lowest density for each variable.

**Table 5:** Built environment variables by cluster

	Cluster	1	2	3	4	5	Total
		Urban neighborhood centers	High employment and population density	High employment and high mixed-use	Urban neighborhoods	High single-family residential	
Population density (persons/sq. mi.)	Mean	11,865.74	6345.35	6023.49	4224.64	2224.34	3542.57
	SD	3944.82	1538.55	3658.35	2566.36	1978.49	3214.37
Employment density (persons/sq. mi.)	Mean	2996.29	85,375.52	17,351.76	1591.22	477.29	1578.93
	SD	4430.57	12,565.77	8409.99	1754.69	968.83	5382.66
Land-use entropy (1.0 is highest entropy)	Mean	0.69	0.75	0.79	0.72	0.61	0.66
	SD	0.13	0.08	0.11	0.11	0.12	0.13
Intersection density (intersections/sq. mi.)	Mean	311.84	454.54	185.38	82.14	43.72	75.33
	SD	171.51	232.70	123.98	52.31	37.22	86.96
Percentage of single detached housing (single detached homes/sq. mi.)	Mean	42.78	2.93	22.67	43.87	87.51	67.25
	SD	27.27	5.11	20.48	20.89	13.87	28.76

A *χ*^2^ test was conducted between these five clusters and the response locations by integration group. Results in Table 6 show that 80.4% of the difference can be explained by the two integration groups. People who integrate green transportation modes are more likely to live in areas with high densities of employment and population or in urban residential neighborhoods (Cluster 2 and Cluster 4). People who do not integrate tend to live in other three types of neighborhoods. While this result is not statistically significant at the 0.05 level, it points to a relation between the built environment and how people use transportation. The easier it is for a person to integrate green transportation modes, there is likely a good chance that they live in a neighborhood with access to all those modes together. But the modes are not yet fully integrated, the correlation is not strong, however, revealing potential for the built environment to encourage FLM connections.

**Table 6:** Integration group by cluster

Integration group	Cluster (*n* and % within group)											*p* value
	1		2		3		4		5		Total	
	19	24.05	1	0.01	7	0.089	38	49.37	14	17.72	79	0.196
No Integration Group	48	28.24	0	0.00	18	10.59	61	35.88	43	25.29	170	0.196
Total	67		1		25		99		57		249	

#### Green Transportation Facilities

**Fig. 4:**
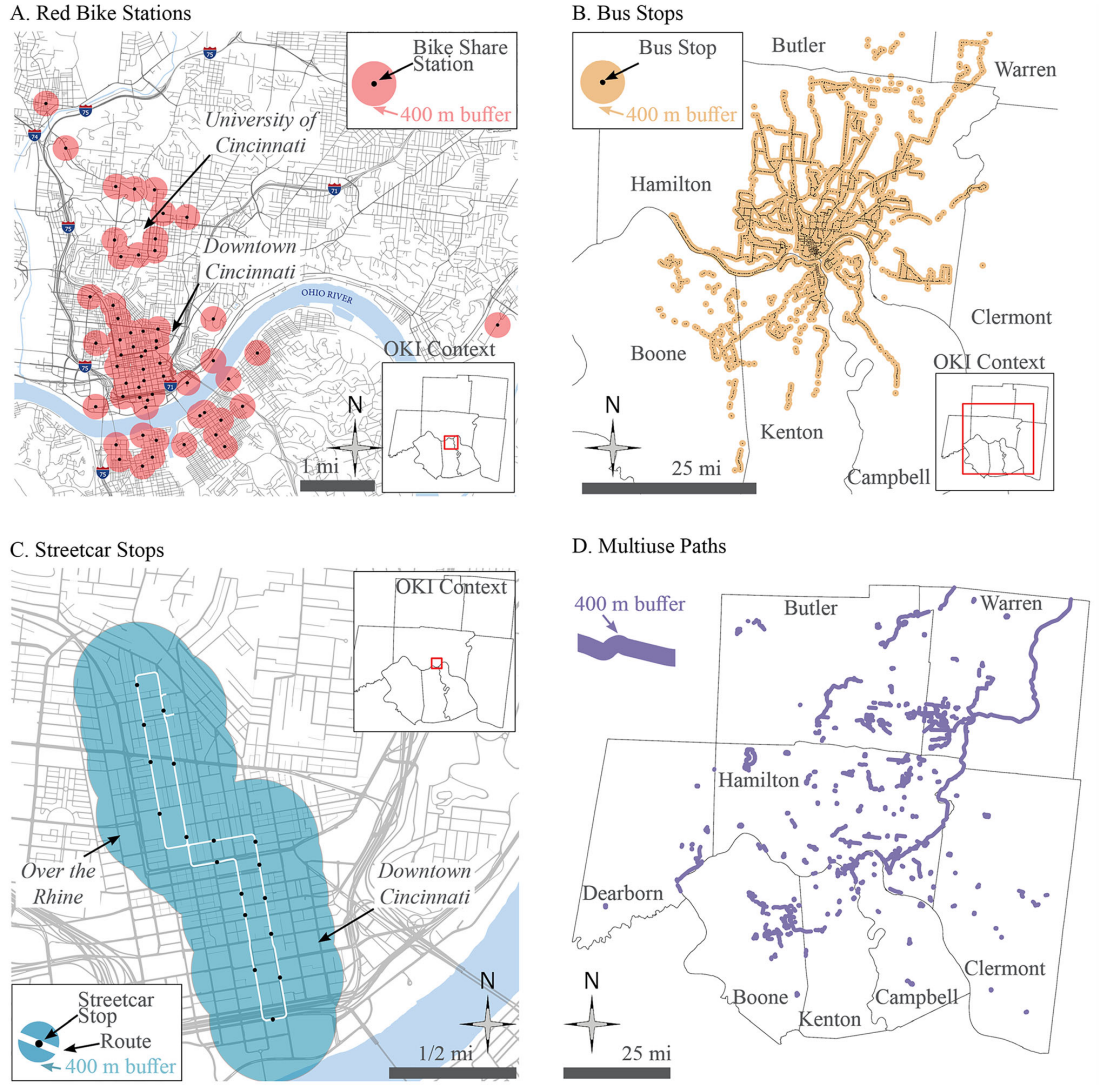
400-Meter buffers

As shown in Fig. 4, bike share, MUPs, and two types of transit service (bus and streetcar) are mapped and vary in terms of coverage area size and distribution throughout the region. The location selections attempt to capture attention and ridership from nearby green transportation facilities. This is consistent with Wang et al. [27]’s report that “…adding bike sharing stations around bus stops increases bike share usage during rush hours.” Understanding how well these facilities are integrated or not will indicate how to improve the network. The more integrated our network is, the better planners can coordinate circulation of people, especially during high-need rush hours.

Geographic mapping of the green transportation 400-meter buffers in Fig. 4 shows a spatial relation to people’s reported level of integration in Fig. 2. Circles were drawn in Fig. 2 to show how in the Integration group, these home locations are close within the urban core and nearest transit, MUPs and bike share. Respondents who were in the Integration Group were found to be in areas with dense street patterns and population, and a low proportion of single detached homes. The proximity to green transportation modes positively influences the likelihood of someone integrating green transportation modes.

When asked of the purpose of their bike share trip along a MUP, 14% reported “commuting,” with other purposes including “recreation” (the highest percentage), “exercise” (also 14%), and “other” (2%). These statistics can be paired with the spatial analysis to decide when and where to improve or add new MUPs to help increase bike share ridership for commuting. These results reveal that TOD strategies could be supported by the overall “overlapping” analysis of the spatial service areas among these green transportation facilities. On the other hand, the service areas of these facilities could be better connected through the establishment of multiple TODs across communities in Cincinnati.

## Discussion and Conclusion

This study examined the barriers to integrating bike share and transit to solve the first/last mile (FLM) issue. Our analysis provides rich evidence through statistical tests and spatial analysis to confirm that people’s integration of green transportation modes is indeed somehow related to their personal and built environment characteristics, both objectively and subjectively. Based on the three research questions, corresponding results and implications for urban and transportation planning are summarized and discussed below.

First, the major barrier to integrating different green transportation modes and/or facilities (bike share, MUPs, public transit) to address the FLM issue lies in the availability and connection between those different green transportation modes and facilities. Attitude-related analysis results by integration level indicate respondents in the study region would be willing to integrate modes more often than they currently do. Specifically, increasing the availability and connection between modes by re-allocating and/or building bike share stations, transit stops, and MUPs closer together would help to improve the barrier of using MUPs as a solution for the FLM issue. It demonstrates the demand for integrating MUPs with bike share and transit to a much greater extent, but also that the distance between MUPs and bike share stations is too far compared with the current demand. There are opportunities to improve the current green transportation network in Greater Cincinnati and a clear demand to do so.

Second, the results of spatial analysis along with the cluster analysis of the built environment show that the location of a person’s home influences their level of integration. The more green transportation facilities a person is within 400 meters of, the more likely that person is to integrate those modes in one journey. This finding obviously points to the importance of spatial proximity and connectivity for promoting the integration of green modes. While a distance of 400 meters was selected based on the general observation of the willingness to bike to a transit stop, more analysis could be done in the future to identify the threshold sensitivity of “proximity” and “connectivity” to help urban planners develop a more spatially efficient green transportation network. While the comparison results between two groups based on the clustering types for the built environment (including intersection density, population density, proportion of single detached homes, employment density, and land-use entropy) are not statistically significant, the comparison direction can still provide some information for the relationship. This limited finding might be improved with more accurate data on responses to the spatial location from the survey respondents.

The major findings summarized above produce recommendations for those stakeholders who are responsible for designing, maintaining, and promoting green transportation facilities and services. One major recommendation this study gives to all parties is better planning and coordination of integrating bike share and MUPs into public transportation system within the framework of TOD strategies. In other words, the address of FLM issue through using the two related solutions, bike share and MUPs, would enhance the technical and political feasibility of TODs across communities by creating a connected and expanded network with some existing resources. This provides a perspective beyond traditional TOD to indicate the function and importance of integrating green transportation facilities and services. While full integration of green transportation modes is currently not financially possible for some medium U.S. cities like Cincinnati, the opportunity for such changes can be considered in the comprehensive plan.

While much effort was made to collect, present, and analyze the data in the best way possible, there are still a few limitations to this study, such as the exclusion of the Cincinnati Bell Connector streetcar in the survey and the omission of asking to clarify what “other” meant in survey questions. Of particular importance, the survey in this study was conducted in October 2020 during the COVID-19 pandemic that prevented in-person outreach at Red Bike stations and when people might have been overwhelmed by emails as so much of normal work for many people went remote. Another limitation is that this study did not examine the relationship between integration level and potential factors with statistical models (e.g., binary logit) as we focus on the descriptive results of the survey and group comparison regarding integration level.

There are several directions this study could be expanded to be more comprehensive and thorough for U.S. cities in order to address challenges in integrated green transportation networks. From March 2020, people began to change where and how they work due to the COVID-19 pandemic. Decreased importance of “getting to work on time” could decrease stress and help people become more open to trying new ways of getting around, such as micro-mobility. How the FLM issue could be addressed with MUPs needs to consider the changes in travel patterns due to the change in living and commute patterns during the COVID-19 pandemic. For instance, studying electric scooters and their connection to other forms of transit would be a direct offshoot of this study. Yet another area of further research could dive deeper into how multiuse paths would contribute to equity and/or accessibility issues, such as where multiuse paths are constructed and what the income level around them is and who uses them. Research to understand how people with different socioeconomic backgrounds access and use MUPs would be helpful for examining the constraints and opportunities of integrating bike paths into the whole green transportation system as initiated in this study.

Integrating bike share and bus/streetcar transit is a sustainable way to increase efficiency in transportation networks, decrease dependency on automobiles, help people become more physically and mentally healthy, and provide more options to transportation-disadvantaged groups. Although there are great examples of how green transportation modes can be integrated, MUPs and people’s perceptions towards their value in the integration have been neglected in the existing planning research concerning the FLM issue. This paper found through an online survey and spatial analysis that MUPs do have the potential to help bike share and transit integration. These results imply that if the status quo is maintained and planning efforts are not expanded, the assets we have invested in, namely the streetcar, bus network, MUPs, and bike share network will not reach their potential in circulating people and thus improving people’s quality of life.

There are existing and growing transportation modes that need to be further integrated and optimized. Attention to the momentum that urban regions already have should manifest in policies that direct funding to integrating green transportation modes. As generations’ beliefs evolve concerning how transportation affects their daily life, plans and policies need to reflect the changes and encourage a seamless transition to integrated green modes of transportation for all users. The well-being of people, cities, and of the environment depend upon it.

## Data Availability

The datasets used in this study are available from the corresponding author on reasonable request.
